# Changes in the Acetylome and Succinylome of *Bacillus subtilis* in Response to Carbon Source

**DOI:** 10.1371/journal.pone.0131169

**Published:** 2015-06-22

**Authors:** Saori Kosono, Masaru Tamura, Shota Suzuki, Yumi Kawamura, Ayako Yoshida, Makoto Nishiyama, Minoru Yoshida

**Affiliations:** 1 Biotechnology Research Center, the University of Tokyo, Bunkyo-ku, Tokyo, Japan; 2 RIKEN Center for Sustainable Resource Science, Wako, Saitama, Japan; University of Freiburg, GERMANY

## Abstract

Lysine residues can be post-translationally modified by various acyl modifications in bacteria and eukarya. Here, we showed that two major acyl modifications, acetylation and succinylation, were changed in response to the carbon source in the Gram-positive model bacterium *Bacillus subtilis*. Acetylation was more common when the cells were grown on glucose, glycerol, or pyruvate, whereas succinylation was upregulated when the cells were grown on citrate, reflecting the metabolic states that preferentially produce acetyl-CoA and succinyl-CoA, respectively. To identify and quantify changes in acetylation and succinylation in response to the carbon source, we performed a stable isotope labeling by amino acids in cell culture (SILAC)-based quantitative proteomic analysis of cells grown on glucose or citrate. We identified 629 acetylated proteins with 1355 unique acetylation sites and 204 succinylated proteins with 327 unique succinylation sites. Acetylation targeted different metabolic pathways under the two growth conditions: branched-chain amino acid biosynthesis and purine metabolism in glucose and the citrate cycle in citrate. Succinylation preferentially targeted the citrate cycle in citrate. Acetylation and succinylation mostly targeted different lysine residues and showed a preference for different residues surrounding the modification sites, suggesting that the two modifications may depend on different factors such as characteristics of acyl-group donors, molecular environment of the lysine substrate, and/or the modifying enzymes. Changes in acetylation and succinylation were observed in proteins involved in central carbon metabolism and in components of the transcription and translation machineries, such as RNA polymerase and the ribosome. Mutations that modulate protein acylation affected *B*. *subtilis* growth. A mutation in acetate kinase (*ackA*) increased the global acetylation level, suggesting that acetyl phosphate-dependent acetylation is common in *B*. *subtilis*, just as it is in *Escherichia coli*. Our results suggest that acyl modifications play a role in the physiological adaptations to changes in carbon nutrient availability of *B*. *subtilis*.

## Introduction

Nε-lysine acetylation is an evolutionarily conserved post-translational modification. Until recently, only a few examples of acetylated proteins had been described in bacteria [1,2]. However, recent proteomic studies on diverse bacteria have identified hundreds of acetylated proteins that function in various cellular processes [3–15]. The addition or removal of an acetyl group from a lysine residue can modify enzymatic activity, protein-protein or protein-DNA interactions, protein stability, or protein localization [16–20]. Lysine acetylation is widely thought to be a reversible process that is catalyzed by two types of enzymes, lysine acetyltransferases (KATs) and lysine deacetylases (KDACs) [21–24] However, two distinct mechanisms for acetylation have been proposed. The first mechanism is KAT-dependent; it utilizes acetyl-CoA as the acetyl group donor. The second mechanism is non-enzymatic. Recent studies have shown that acetyl-CoA serves as the acetyl donor in the mitochondria [25], while acetyl phosphate (acetyl-P) serves as the acetyl donor in bacteria [9,12]. Acetylation in *Escherichia coli* commonly uses acetyl-P as the acetyl donor [9,12]. CobB, the only known KDAC in *E*. *coli*, can deacetylate both enzymatic and non-enzymatic lysine acetylation substrates, but not all acetylation substrates, suggesting that some of acetylations cannot be reversed [26].

Recent advances in mass spectrometry have facilitated the discovery of new acyl modifications of lysine residues, including propionylation, butyrylation, succinylation, malonylation, crotonylation, and glutarylation [27–31]. Of these, propionylation, succinylation, and malonylation have been reported in bacteria [28,29,32–34]. It is thought that the corresponding acyl-CoA molecule is utilized in these acyl modifications. In bacteria, acetyl-CoA and succinyl-CoA are abundant and are mainly generated from glycolysis and the citrate cycle, respectively [35]. Lysine succinylation and acetylation are frequent modifications in bacteria and eukarya [36]. Hundreds of succinylated proteins have been reported in *E*. *coli* [33,36], and *E*. *coli* CobB, catalyzes not only deacetylation, but also desuccinylation [33]. However, an enzyme that catalyzes lysine succinylation has not yet been identified.

In *Bacillus subtilis*, AcuA, the only known KAT, contains a Gcn5-related acetyltransferase (GNAT) motif. AcuA is not a close homologue of YfiQ (also known as Pka or PatZ in *E*. *coli*; Pat in *Salmonella*), the only known KAT in *E*. *coli*. *B*. *subtilis* has two KDACs, AcuC and SrtN, which are NAD^+^-independent and NAD^+^-dependent (sirtuin family) deacetylases, respectively. AcuA, AcuC, and SrtN control the enzymatic activity of acetyl-CoA synthetase (AcsA) through the reversible acetylation of a conserved, critical lysine residue, Lys549 [37–39]. A recent acetylome analysis revealed 185 acetylated proteins in *B*. *subtilis* [7]. However, a comprehensive analysis of lysine succinylation in *B*. *subtilis* has not yet been reported.

Increasing evidence indicates that acyl modifications contribute to the control of metabolic enzymes in bacteria and eukarya [2,5,20,21,24,40]. In most central pathways for carbon metabolism in *B*. *subtilis*, oscillations in enzyme concentration are insufficient to explain changes in metabolic flux, and allosteric regulation and enzyme modification have emerged as important mechanisms that control metabolic enzymes and fluxes [41]. Acetylation and succinylation are closely linked to central carbon metabolism through glycolysis and the citrate cycle, respectively. Because many metabolic and regulatory networks have been studied extensively in *B*. *subtilis* [41–43], we thought that the organism would be suitable for examining the effect of acyl modifications on carbon flux regulation. In this study, we used a quantitative, proteomic approach based on stable isotope labeling by amino acids in cell culture (SILAC) to identify and quantify the changes in lysine acetylation and succinylation in response to the carbon source, with glucose and citrate as glycolytic and citrate cycle substrates, respectively. Our study revealed that, the two acyl modifications changed in response to the carbon source in *B*. *subtilis*. Mutations that modulate protein acylation affected *B*. *subtilis* growth in a carbon source-dependent manner. The possible role of acyl modifications in the physiological responses and adaptations to changes in carbon nutrients is discussed.

## Materials and Methods

### Bacterial strains and culture conditions

*B*. *subtilis* strain 168 (*trpC2*), obtained from the *Bacillus* Genetic Stock Center (BGSC 1A1), was used as the wild type strain in this study. To construct a lysine auxotroph strain for SILAC, the codons for Arg8 (AGA) and Gln9 (CAA) in the *lysA* gene were replaced with nonsense mutations. To accomplish this, oligonucleotide primers were used to amplify the upstream (lysAmut1-F and lysAmut1-R) and downstream (lysAmut2-F and lysAmut2-R) regions of the *lysA* gene (see S1 Table for the nucleotide sequences of all primers used in this study). Another PCR was performed using the primers lysAmut1-F and lysAmut2-R and the above two amplified fragments as the DNA template to generate a mutation-containing fragment. We also amplified the *trpC2*-*hisC* region by PCR using the primers trpC2hisC-F and trpC2hisC-R and the chromosomal DNA of strain 168 as the template. The resulting two PCR fragments, a *lysA* mutation (*lysA8*)-containing fragment and a *trpC2*-*hisC* fragment, were used to transform strain RIK1800 (168 *hisC101*) [44], and histidine-autotroph transformants were selected. Tryptophan-auxotroph transformants with a *trpC2* genetic background were further selected, and the resulting strain was designated strain TM61 (168 *trpC2 lysA8*). To construct deletion mutants of *acuA*, *acuC*, *srtN*, *ackA*, and *pta*, the upstream and downstream regions (approximately 0.6~1.0 kbp in length) of each target gene were amplified by PCR using the corresponding del1-F and del1-R primers and del2-F and del2-R primers, respectively (see S1 Table). A 1.2 kb-erythromycin resistance gene cassette was excised from pAE41 [45] through *Bam*HI and *Sph*I digestion and ligated with the upstream and downstream fragments of *acuA*, resulting in an *acuA*::*erm* fragment. A 1.3-kb neomycin resistance gene cassette was excised from pBEST501 [46] through *Xba*I digestion and ligated with the upstream and downstream fragments of *acuC*, resulting in an *acuC*::*neo* fragment. A 1.4-kb spectinomycin resistance gene cassette was excised from pBEST517A [47] through *Xba*I digestion and ligated with the upstream and downstream fragments of *srtN*, resulting in an *srtN*::*spc* fragment. A 0.97-kb spectinomycin resistance gene cassette was amplified by PCR using primers ackA_spc-2F and ackA_spc-2R with pBEST517A as a template; the product was connected by splicing by overlap extension (SOE)-PCR with the upstream and downstream fragments of *ackA*, resulting in an *ackA*::*spc* fragment. A 0.89-kb kanamycin resistance gene cassette was amplified by PCR using primers pta_kan-F and pta_kan-R with RIK1420 [48] as a template; the product was connected by SOE-PCR with the upstream and downstream fragments of *pta*, resulting in a *pta*::*kan* fragment. The resulting *acuA*::*erm*, *acuC*::*neo*, *srtN*::*spc*, *ackA*::*spc*, and *pta*::*kan* fragments were used to transform strain 168. The transformants that harbored a deletion mutation in the target gene as a result of a double-crossover event were selected for resistance to erythromycin (0.5 μg/ml), spectinomycin (50 μg/ml), neomycin (7.5 μg/ml), or kanamycin (5 μg/ml) at 37°C. Proper disruption of each target gene was confirmed by PCR amplification and DNA sequencing. Finally, strain SS38 (*acuC*::*neo srtN*::*spc*), SS51 (*ackA*::*spc*), SS52 (*pta*::*kan*), SS53 (*ackA*::*spc pta*::*kan*), SS110 (*acuA*::*erm*), and SS111 (*ackA*::*spc pta*::*kan acuA*::*erm*) were obtained, as listed in Table 1.

**Table 1:** *Bacillus subtilis* strains used in this study.

Strain	Relevant characteristic	Source/reference
168	*trpC2*	BGSG 1A1
TM61	168 *lysA8*	This study
SS38	168 *acuC*::*neo srtN*::*spc*	This study
SS51	168 *ackA*::*spc*	This study
SS52	168 *pta*::*kan*	This study
SS53	168 *ackA*::*spc pta*::*kan*	This study
SS110	168 *acuA*::*erm*	This study
SS111	168 *ackA*::*spc pta*::*kan acuA*::*erm*	This study

For western blotting, wild type strain 168 cells were grown in modified Spizizen minimal medium (0.6 g/l KH_2_PO_4_, 1.4 g/l K_2_HPO_4_, 0.2 g/l (NH_4_)_2_SO_4_, 1 mM MgSO_4_, 1 mM CaCl_2_, 10 μM MnCl_2_, and 1 μM FeSO_4_) supplemented with 50 μg/ml tryptophan, 0.01% yeast extract, and a carbon source (30 mM). They were harvested at OD_660_ = 0.5–0.7. For SILAC labeling, strain TM61 colonies grown on a minimal medium plate supplemented with 30 mM glucose, a 50 μg/ml amino acid mixture (without lysine), and 50 μg/ml ^13^C_6_-lysine (heavy lysine) were inoculated into fresh minimal medium supplemented with glucose and heavy lysine at OD_660_ = 0.1. They were grown until the OD_660_ reached 0.5 for pre-cultivation. For glucose-heavy labeling (exp. 1), the cultures were grown in fresh minimal medium supplemented with glucose and heavy lysine or in minimal medium supplemented with citrate and light lysine. For citrate-heavy labeling (exp. 2), the cultures were grown in minimal medium supplemented with glucose and light lysine or in minimal medium supplemented with citrate and heavy lysine. The cultivation was started at OD_660_ = 0.1, and the cells were harvested at OD_660_ = 0.5.

### Preparation of a pan-anti-succinyllysine antibody

A pan-anti-succinyllysine antibody against succinylated bovine serum albumin (BSA) was generated as previously described [49]. BSA was chemically succinylated with succinic anhydride, and the degree of chemical succinylation of primary amines was confirmed by MALDI-TOF MS (76% of the amino groups were succinylated). The rabbit polyclonal anti-succinyllysine antiserum was generated by the Research Resource Center (RRC) of RIKEN-BSI. Specificity of the homemade pan anti-succinyl lysine antibody was confirmed by dot blot analyses (S1 Fig).

### Preparation of protein lysates and western blot analysis

Cells were lysed in NET buffer (50 mM Tris-HCl, pH 7.6, 150 mM NaCl, and 1 mM EDTA) supplemented with 1 mM DTT, 1 mM PMSF, 10 μg/ml DNase, 10 μg/ml RNase, 10 mM sodium butyrate (class-I, II KDAC inhibitor), and 20 mM nicotinamide (class-III or sirtuin KDAC inhibitor) by exposure to high pressure using an EmulsiFlex-B15 (Avestin). After removing the cell debris by centrifugation, the cleared lysates were concentrated with a Vivaspin 20 (Sartorius). The protein concentration was measured with the Quick start protein assay (Bio-Rad). Lysate aliquots containing 20 μg of protein were separated by 10% SDS-PAGE and then transferred to an Immobilon-P membrane (Millipore) using a semidry apparatus. The blot was blocked with 3% (w/v) skim milk in TBST, and then incubated with a mixture of rabbit polyclonal antibodies (1:1000 each in 3% [w/v] milk-TBST) as the primary antibody at 4°C overnight. The blot was then incubated with an horseradish peroxidase-conjugated goat anti-rabbit secondary antibody (1:5000 in 3% milk-TBST; Sigma) for 1 h at room temperature. Antigens on the membranes were detected with an LAS4000 image analyzer (GE Healthcare).

### Preparation of protein lysates, tryptic digestion, and enrichment of lysine-acetylated and-succinylated peptides

Equal amounts of protein (2.5 mg of protein from the glucose and citrate conditions) were mixed, precipitated with acetone, and dissolved in 0.1 M NH_4_HCO_3_. Proteins were reduced with 20 mM DTT at 56°C for 30 min, and subsequently alkylated with 30 mM iodoacetamide at 37°C for 30 min. Samples were incubated overnight at 37°C with sequencing grade trypsin (Promega) at a 1:100 enzyme:substrate ratio (w/w). Proteolytic peptides were concentrated by vacuum centrifugation, and then resuspended in NETN buffer (50 mM Tris-HCl, pH 7.6, 150 mM NaCl, 1 mM EDTA, and 0.1% NP-40). A mixture of polyclonal anti-acetyllysine antibodies (Cell Signaling and Rockland) or the anti-succinyllysine antibody (this study) was added at a 1:100 antibody:peptide ratio (w/w) to collect the acetylated or succinylated peptides, respectively. The acetylated and succinylated peptides captured by the respective antibodies were precipitated with protein-G beads (Veritas). The beads were washed three times in NETN buffer and twice in NET buffer. The enriched acetylated peptides were then eluted with 1% trifluoroacetic acid:50% acetonitrile. The acetylated peptide samples were cleaned with ZipTip-scx (Millipore) according to the manufacturer’s instructions, and then subjected to HPLC-MS/MS analysis. To compare the relative abundance of proteins in the glucose and citrate conditions, equal amounts of protein from the two growth conditions were mixed, reduced, alkylated, digested with trypsin, and subjected to MS analysis. The labeling efficiency was calculated as the amount of light peptides among the total peptides in labeled samples without affinity enrichment.

### Mass spectrometry analysis, peptide identification, and SILAC quantification

Samples were analyzed by reverse-phase nano HPLC-MS/MS using an EASY-nLC 1000 HPLC system (Thermo Fisher Scientific) connected to a Q Exactive mass spectrometer (Thermo Fisher Scientific). Peptides were trapped on an Acclaim PepMap pre-column (100 μm × 2 cm; Thermo Fisher Scientific) and then eluted onto a Nano-HPLC capillary C18 column (0.075 × 150 mm; Nikkyo Technos). They were then separated with a 120-min gradient from 0% to 65% solvent B (0.1% formic acid in acetonitrile; solvent A, 0.1% formic acid) at a flow rate of 300 nl/min. Full scan MS spectra (350–1,800 *m*/*z*) were acquired in the Orbitrap with a target value of 3.00E+06 at a resolution of 70,000 at 200 *m*/*z*. The 10 most intense ions were selected for higher-energy-collisional-dissociation (HCD) fragmentation in the HCD cell with a target value of 1.00E+05 and normalized collision energy of 28%.

Mass spectrometric data were processed using Proteome Discoverer (ver. 1.4; Thermo Fisher Scientific). Data were searched against *B*. *subtilis* sequences (4,185 entries) in the SwissProt database (2013_11) using with the MASCOT search engine (ver. 2.4.1). The search parameters in MASCOT included trypsin digestion with two missed cleavages allowed. Variable modifications included ^13^C_6_-lysine, lysine acetylation, lysine succinylation, and methionine oxidation; cysteine carbamidomethylation was set as a fixed modification. Precursor ion and fragment ion mass tolerances were set to 6 ppm and 20 mmu, respectively. For both identification and quantification, only spectra with expectation values less than 1% of the false discovery rate (FDR) were accepted. Identified peptides were validated using the Percolator algorithm with a q-value threshold of 0.01. The identified peptides with Mascot ion scores below 20 were removed to ensure high quality peptide identification. The event detector and precursor ion quantifier algorithms of Proteome Discoverer were used for quantification with a 2 ppm mass variability and a 0.2 min retention time tolerance on precursor ion pairs. Quantification was based on the ratio of the peak area for each peptide in the glucose and citrate conditions. The peptide ratios were calculated using the same number of isotopes. Protein ratios were calculated using the total trypsinized peptides without affinity enrichment based on a previously reported method [50]. The mass spectrometry proteomics data have been deposited with the ProteomeXchange Consortium (http://proteomecentral.proteomexchange.org) via the PRIDE partner repository [51] (dataset PXD001615).

The change in acyl modification was determined from the R-value, which was calculated from the ratio of the peptide peak areas (Heavy/Light [pep]) normalized to the ratio of the protein areas (Heavy/Light [pro]). When multiple peptides with a single lysine modification were detected, the peptide with the highest ion score was chosen. Comprehensive lists of the unique acetylated and succinylated peptides identified are provided with the R-values in S2 Table (for each experiment) and S3 Table (merged).

### Bioinformatics analysis

For protein function annotation, we used the Kyoto Encyclopedia of Genes and Genome (KEGG) pathway database with BRITE functional hierarchies. For functional enrichment analysis, we used the Database for Annotation, Visualization, and Integrated Discovery (DAVID) [52]. A Bonferroni cutoff of 0.05 was used to determine the statistical significance. For motif analysis, the 10 amino acid residues (-10 to +10) on either side of a modification site were selected, and a consensus logo was generated using the iceLogo webserver [53]. We also analyzed the same sets of data using the motif-X webserver [54] to confirm the sequence preferences (S4 Fig). All information for the protein annotations, the positions of acyl modifications, and their surrounding sequences are shown in S2 Table.

## Results

### Changes in acyl modifications in *B*. *subtilis* in response to the carbon source

**Fig 1:**
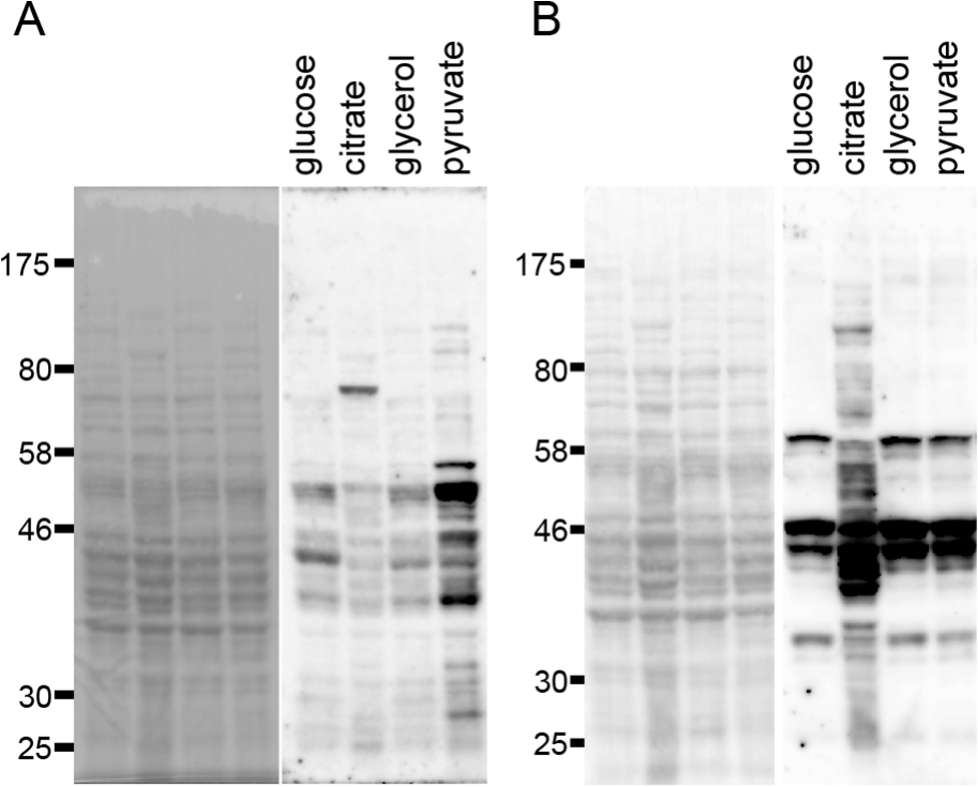
Changes in *B*. *subtilis* lysine acetylation and succinylation in response to different carbon sources. Cells were grown in modified Spizizen’s minimal medium supplemented with glucose, citrate, glycerol, or pyruvate (30 mM) as the sole carbon source. Total lysates containing 20 μg of protein were separated by 10% SDS-PAGE. Left: Ponceau staining; right: western blot with anti-acetyl lysine (A) and anti-succinyl lysine (B) antibodies.

Changes in carbon metabolic flux depend on the carbon source used for growth. Glycolysis occurs when cells are grown on glucose, whereas gluconeogenesis and the citrate cycle are activated to supply building blocks when cells are grown on citrate, a citrate cycle substrate. Both glycolysis and gluconeogenesis are activated when cells are grown on glycerol, and the citrate cycle is more active in cells grown on pyruvate than in cells grown on glycerol [41]. To investigate the correlation between carbon metabolism and acyl modification, we examined global acetylation and succinylation using western blot analysis in cells grown on several different carbon sources (Fig 1). Acetylation occurred when the cells were grown on pyruvate, glucose, glycerol, and citrate, in decreasing order, as the sole carbon source (Fig 1A). In addition, acetylation patterns were different when the cells were grown in the presence of glucose, citrate, and pyruvate. In contrast, succinylation was higher in cells grown on citrate than in cells grown on the other carbon sources (Fig 1B). The succinylation pattern in the presence of citrate was also different from that in the presence of other carbon sources. These patterns of acyl modification seemed to reflect the levels of acetyl-CoA and succinyl-CoA, which are generated by pyruvate dehydrogenase via glycolysis and 2-oxoglutarate dehydrogenase in the citrate cycle, respectively. The results indicated that acetylation and succinylation change in response to the carbon source in *B*. *subtilis*. The specificities of pan anti-acetyl lysine and anti-succinyl lysine antibodies were determined by dot blot analyses (S1 Fig).

### Changes in the *B*. *subtilis* acetylome in response to the carbon source

**Fig 2:**
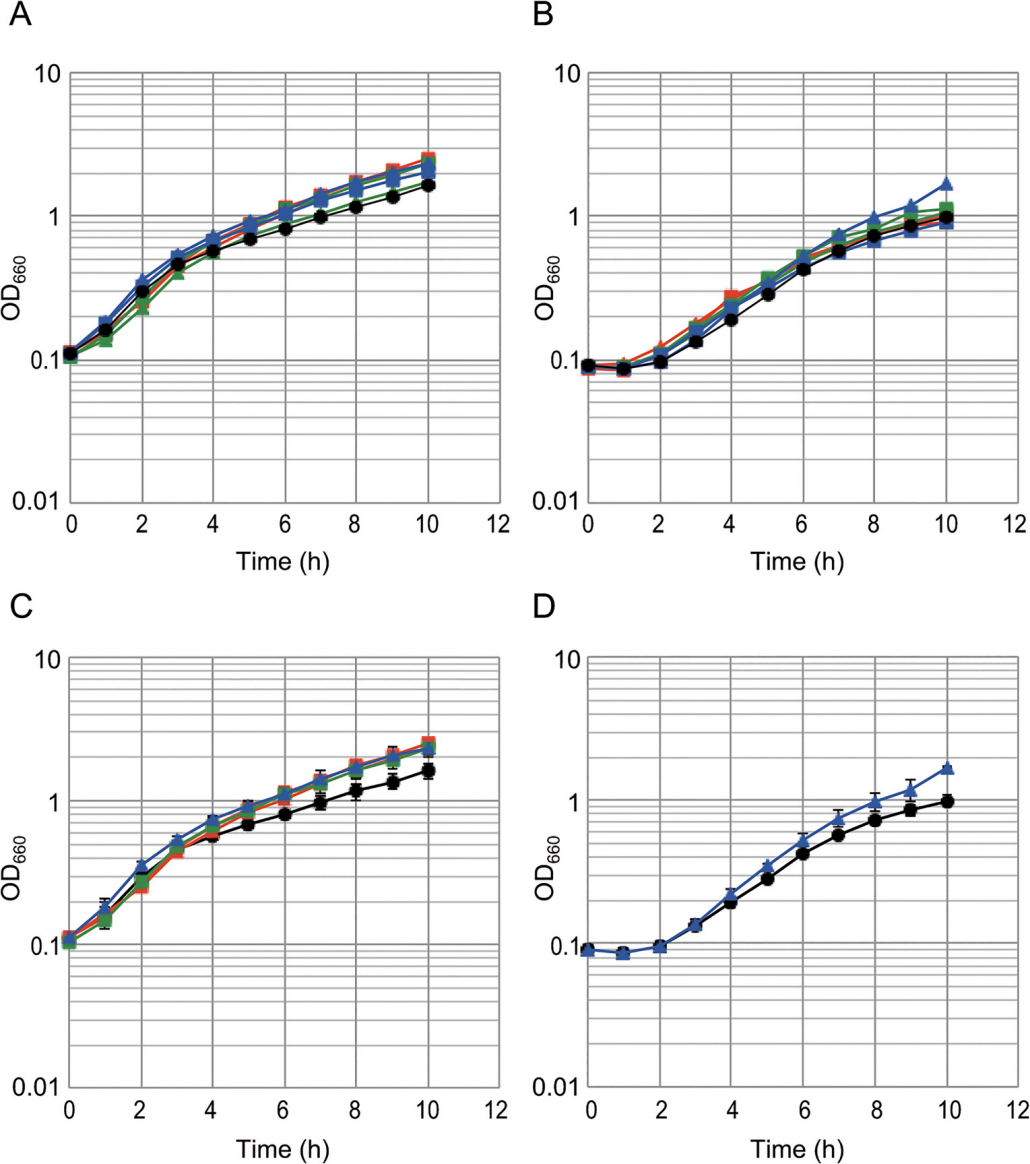
Growth of wild type and mutant strains in glucose or citrate. Fresh colonies grown on minimal glucose plates supplemented with amino acid mixture were inoculated in a modified Spizizen’s minimal medium supplemented with 30 mM glucose (A and C) or 30 mM citrate (B and D). Growth curves were monitored by measuring OD at 660 nm. 168 (WT, black circle), SS110 (Δ*acuA*, blue triangle), SS38 (Δ*acuC* Δ*srtN*, blue square), SS51 (Δ*ackA*, green triangle), SS52 (Δ*pta*, green square), SS53 (Δ*ackA* Δ*pta*, red triangle), and SS111 (Δ*ackA* Δ*pta* Δ*acuA*, red square) strains. Panels A and B show the WT and isogenic mutant strains plotted along with the average OD_660_ values from three growth experiments. Panel C shows SS52 (Δ*pta*), SS53 (Δ*ackA* Δ*pta*), SS110 (Δ*acuA*), SS111 (Δ*ackA* Δ*pta* Δ*acuA*), and WT strains along with error bars, as reproduced from panel A. Panel D shows SS110 (Δ*acuA*) and WT strains along with error bars, as reproduced from panel B.

To evaluate the changes in lysine acetylation and succinylation in response to the carbon source, we used a quantitative MS approach based on SILAC. We chose glucose or citrate as the different growth conditions, because we observed different acyl modification patterns with these substrates, as shown in Fig 1. Under these two conditions, the carbon metabolic fluxes are totally different [41]. We chose an OD_660_ of 0.5 as the growth point at which wild type cell growth was exponential (Fig 2). For glucose-heavy labeling (exp. 1), a *B*. *subtilis* lysine auxotroph strain (TM61) was grown in glucose with heavy lysine medium or in citrate with light lysine medium. For citrate-heavy labeling (exp. 2), the strain was grown in glucose with light lysine medium or in citrate with heavy lysine medium. We confirmed with western blot analysis that the *lysA* mutation in the TM61 strain did not affect the global acetylation and succinylation profiles when compared to those in the wild type strain (S2 Fig). After labeling, lysates from both conditions containing equal amounts of protein were mixed and digested with trypsin. The acetylated or succinylated lysine peptides were then affinity enriched using anti-acetyllysine or anti-succinyllysine antibodies, respectively. We also analyzed the mixed total trypsinized peptides without affinity enrichment to estimate the relative abundance of the proteins under these two conditions. The labeling efficiencies were 99.8% for glucose-heavy labeling and 99.7% for citrate-heavy labeling. The change in modification levels was evaluated by determining the R-value, which was calculated from the ratio of peptide peak areas normalized to the ratio of protein abundance. The data were obtained from duplicate experiments with switched labeling (exp. 1 and exp. 2). The acetylation and succinylation sites identified in each experiment are summarized in S3 Fig.

**Fig 3:**
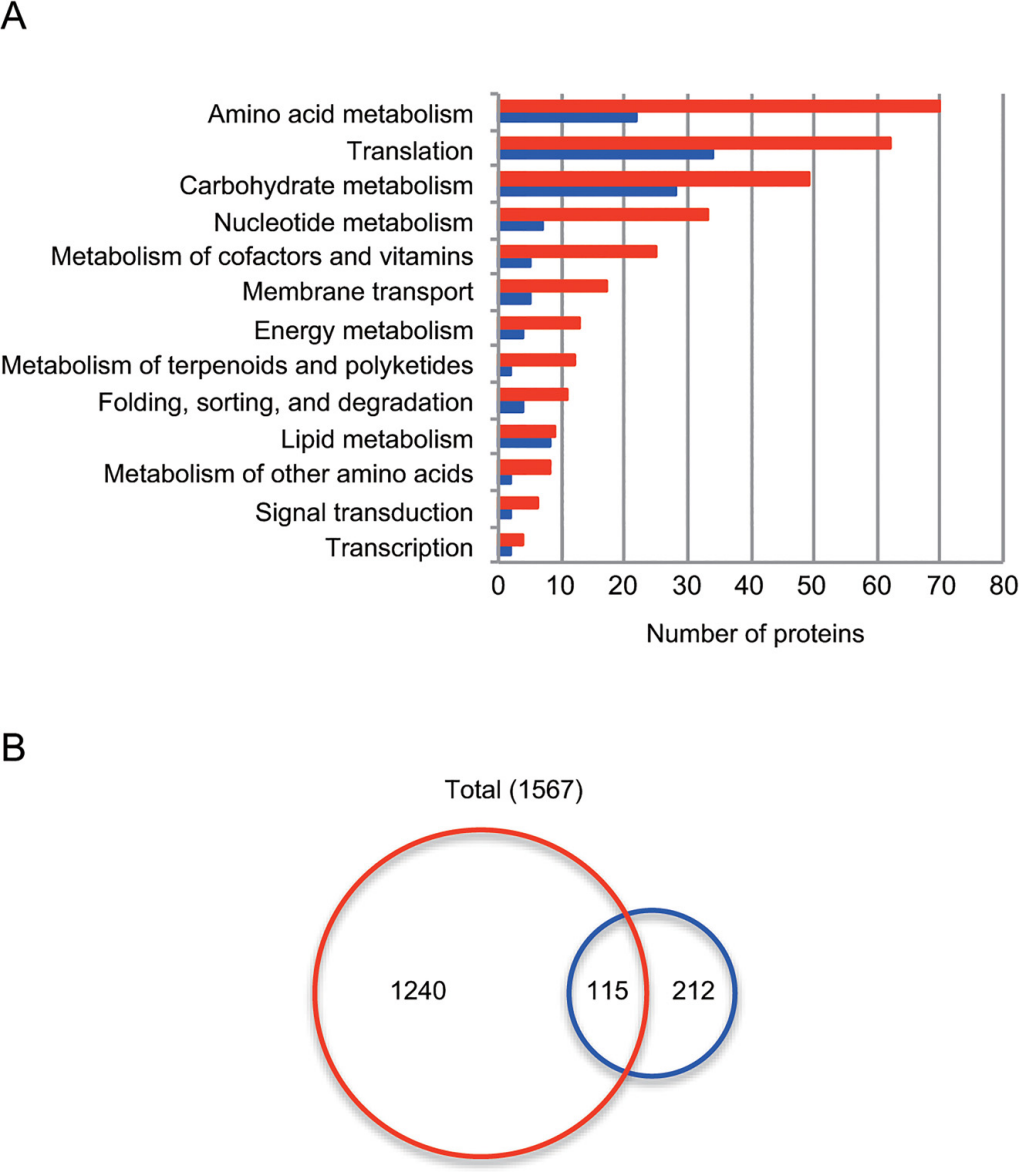
The acetylome and succinylome of *B*. *subtilis* profiled in this study. (A) Functional classification of the identified acetylated proteins (629) and succinylated proteins (204) based on the KEGG pathway database. Red and blue bars represent the number of acetylated and succinylated proteins, respectively. (B) Overlap between acetylation (1355) and succinylation (327) sites. Red and blue circles enclose the number of acetylation and succinylation sites, respectively.

A total of 1355 unique acetyllysine sites were identified on 629 acetylated proteins (Table 2). We performed a functional classification analysis of acetylated proteins based on the KEGG pathway database (Fig 3A). The largest groups contained proteins involved in amino acid metabolism (70 proteins, 11% of the total), followed by proteins involved in translation (62 proteins, 10%) and carbohydrate metabolism (49 proteins, 8%). This profile is similar to a profile previously reported for the *B*. *subtilis* acetylome [7]. The most heavily acetylated proteins included SrfAA (21 sites), SrfAB (21 sites), Tuf (12 sites), PurB (10 sites), and RplB (10 sites).

**Table 2:** The acetylome and succinylome of *Bacillus subtilis*.

	Acetylation sites	Succinylation sites
Reproducible sites	482	43
Reproducibly up-regulated in		
Glucose condition^a^	13	0
Citrate condition^b^	40	42
Proportional^c^	150	0
Others^d^	279	1
Non-reproducible sites	873	284
Total	1355 (in 629 proteins)	327 (in 204 proteins)

^a^Sites with an R-value >2-fold higher in glucose or sites specifically detected in glucose.

^b^Sites with an R-value >2-fold higher in citrate or sites specifically detected in citrate.

^c^Sites with an R-value with a fold change of <2.

^d^Sites that did not show a reproducible change.

To gain further insight into how lysine acetylation regulates cellular function, we performed a pathway enrichment analysis of each condition using DAVID and the KEGG database (Table 3). In both glucose and citrate conditions, acetylated proteins were enriched in translation-related categories, aminoacyl-tRNA biosynthesis and ribosome. Acetylation also preferentially targeted different metabolic pathways under the two conditions: valine, leucine, and isoleucine biosynthesis and purine metabolism in the glucose condition and the citrate cycle in the citrate condition.

**Table 3:** KEGG pathway enrichment for acetylated proteins in the glucose and citrate conditions.

Term^a^	KEGG pathway	Count^b^	%^c^	*p*-value^d^	Fold enrichment^d^	Bonferroni^d^ ^,^ ^e^
***In the glucose condition***						
bsu00970	Aminoacyl-tRNA biosynthesis	21	4.5	4.18E-08	3.2	3.51E–06
bsu00290	Valine, leucine, and isoleucine biosynthesis	14	3.0	1.07E-04	2.9	8.97E–03
bsu03010	Ribosome	40	8.5	7.15E-10	2.5	6.00E–08
bsu00230	Purine metabolism	33	7.0	1.17E-05	2.0	9.81E–04
***In the citrate condition***						
bsu00970	Aminoacyl-tRNA biosynthesis	17	3.3	1.29E-04	2.5	0.0112
bsu00020	Citrate cycle (TCA cycle)	16	3.1	3.22E-04	2.5	0.0277
bsu03010	Ribosome	38	7.4	4.25E-08	2.3	3.70E–06

^a^KEGG pathway term.

^b^Number of genes matching a given KEGG pathway term.

^c^Percentage of genes matching a given term divided by the total number of input genes in each condition.

^d^The whole genome of *B*. *subtilis* was used as background. (An additional analysis performed using “the total *B*. *subtilis* proteins identified by MS from cell lysates” as background resulted in the same list of enrichment categories.)

^e^A Bonferroni cutoff value of 0.05 was used.

Of 1355 acetylated sites, 482 sites were reproducibly detected in the two experiments and used for quantitative analysis. At 13 sites, acetylation was reproducibly upregulated in the glucose condition relative to the citrate condition (Table 4). These sites were found in proteins involved in amino acid metabolism (AroA, AsnB, and IlvK), nucleotide metabolism (PurD, PurF, and PyrAB), and translation (RplP, RpsD, and Tuf). In the citrate condition, acetylation was reproducibly upregulated at 40 sites (Table 4) in proteins involved in carbohydrate metabolism (AckA, AcsA, DhaS, LutA, LutC, and PckA), amino acid metabolism (ArgF, HutI, YodQ, and YcgN), and energy metabolism (CtaC, QcrA, YjlD, and YumB). Interestingly, the siderophore biosynthesis proteins DhbC and DhbF contained acetylation sites that were differentially regulated: acetylation of K145 and K466 was upregulated in glucose, whereas acetylation of K11 and K1522 was upregulated in citrate (Table 4). Unexpectedly, we observed that several sporulation-related proteins (CotE, CotO, Cse15, SpoIVD, and SpoVR) contained upregulated acetylation sites in the citrate condition. We observed spore formation in the citrate condition (3.7 × 10^4^ spores/ml), but not in the glucose condition (less than 10 spores/ml). Upregulation of sporulation genes has been reported during growth on pyruvate or succinate with glutamate [41]. At present, we do not know the biological significance of the increase in acetylation in these sporulation-related proteins. However, slow growth on pyruvate, succinate with glutamate, or citrate might induce responses that mimic starvation, as suggested previously [41]. Of 482 acetylation sites reproducibly detected, 150 had a fold change of <2 (0.5 ≤ R ≤ 2), indicating that the change in the modification level was in proportion to the change in the protein amount (“proportional” in Table 2).

**Table 4:** Acetylation sites upregulated in the glucose or citrate condition^a^.

Protein	Description	Position	R-value (glc to cit)^b^	
			Exp. 1	Exp. 2
***Upregulated in the glucose condition (13 sites)***				
AroA	5-Enolpyruvylshikimate-3-phosphate synthetase	K142	G^c^	2.1
AsnB	Asparagine synthetase	K239	G	G
DhbC	Isochorismate synthase	K145	G	G
DhbF	Dimodular nonribosomal peptide synthase	K466	G	G
IlvK	Branched-chain-amino-acid aminotransferase 2	K42	G	G
PdhA	Pyruvate dehydrogenase E1 component subunit α	K283	G	G
PurD	Phosphoribosylamine-glycine ligase	K396	G	2.1
PurF	Amidophosphoribosyltransferase	K455	G	G
PyrAB	Carbamoyl-phosphate synthase pyrimidine-specific large chain	K395	G	G
RacE	Glutamate racemase 1	K248	G	G
RplP	50S ribosomal protein L16	K77	G	2.2
RpsD	30S ribosomal protein S4	K81	G	44
Tuf	Elongation factor Tu	K42	18	G
***Upregulated in the citrate condition (40 sites)***				
AckA	Acetate kinase	K309	0.10	0.14
AcsA	Acetyl-coenzyme A synthetase	K32	0.34	C
AcsA	Acetyl-coenzyme A synthetase	K320	C^c^	C
AppF	Oligopeptide transport ATP-binding protein	K139	C	C
ArgF	Ornithine carbamoyltransferase	K55	C	C
CotE	Spore coat protein E	K10	C	C
CotO	Spore coat protein O	K37	C	C
Cse15	Sporulation protein cse15	K214	C	C
Cse15	Sporulation protein cse15	K70	C	C
CtaC	Cytochrome c oxidase subunit 2	K196	C	C
CtaC	Cytochrome c oxidase subunit 2	K318	C	C
DhaS	Putative aldehyde dehydrogenase	K10	C	C
DhaS	Putative aldehyde dehydrogenase	K282	C	C
DhbC	Isochorismate synthase	K11	0.0015	0.42
DhbF	Dimodular nonribosomal peptide synthase	K1522	0.0096	0.14
DppE	Dipeptide-binding protein	K354	C	C
EtfB	Electron transfer flavoprotein subunit beta	K67	C	C
EtfB	Electron transfer flavoprotein subunit beta	K50	0.30	C
HutI	Imidazolonepropionase	K130	C	C
LutA	Lactate utilization protein A	K95	C	C
LutC	Lactate utilization protein C	K94	C	C
PckA	Phosphoenolpyruvate carboxykinase [ATP]	K31	C	C
PckA	Phosphoenolpyruvate carboxykinase [ATP]	K375	C	C
PrkA	Protein PrkA	K335	C	C
QcrA	Menaquinol-cytochrome c reductase iron-sulfur subunit	K88	C	C
RplB	50S ribosomal protein L2	K244	0.29	0.39
RplB	50S ribosomal protein L2	K252	0.29	0.38
SpoVID	Stage VI sporulation protein D	K510	C	C
SpoVID	Stage VI sporulation protein D	K514	C	C
SpoVR	Stage V sporulation protein R	K355	C	C
SrfAA	Surfactin synthase subunit 1	K3154	0.40	0.36
SrfAB	Surfactin synthase subunit 2	K1621	0.12	C
YcgN	1-Pyrroline-5-carboxylate dehydrogenase 2	K409	0.46	C
YhaX	Stress response protein	K282	C	C
YjlD	NADH dehydrogenase-like protein	K37	0.0039	C
YkfB	l-Ala-d/l-Glu epimerase	K17	C	C
YkfD	Putative oligopeptide transport ATP-binding protein	K25	C	C
YodQ	Uncharacterized metallohydrolase	K320	C	C
YufN	Uncharacterized lipoprotein	K201	C	C
YumB	NADH dehydrogenase-like protein	K347	C	C

^a^Acetylation sites that were reproducibly changed in the two experiments were chosen.

^b^R-value (the ratio of glucose to citrate) is shown.

^c^G, detected only in the glucose condition; C, detected only in the citrate condition.

We compared our acetylome data with previously reported *B*. *subtilis* acetylome data obtained from a study of stationary phase cells in LB medium [7]. Only 62 sites (5% of our acetylome) were shared by the two acetylomes. The low overlap might be due in part to differences in the experimental setups, including the antibodies and media used, as well as the growth phase of the cells.

We also compared our acetylome data with the *E*. *coli* acetylome data obtained from a study of cells grown in glucose-supplemented minimal medium [9]. We performed a DAVID analysis using the same conditions used in Table 3. We observed enrichment of acetylated proteins in the categories of aminoacyl-tRNA biosynthesis; ribosome; the citrate cycle; purine metabolism; and valine, leucine, and isoleucine biosynthesis (S4 Table). These findings are similar to those obtained with the *B*. *subtilis* acetylome (Table 3), except that proteins involved in the citrate cycle are enriched in minimal glucose condition. We also observed that *E*. *coli* acetylated proteins are more enriched in the categories of fatty acid biosynthesis; histidine metabolism; and phenylalanine, tyrosine, and tryptophan biosynthesis in the minimal glucose condition (S4 Table).

Analysis using the iceLogo and motif-X algorithms indicated that negatively charged residues (D and E) were overrepresented in the regions surrounding the 1355 acetyllysine sites (S4 Fig), as previously reported in the acetylomes of *B*. *subtilis* [7], *Thermus thermophiles* [8], and *E*. *coli* [12,26,55]. The similarity of our acetylome with other acetylomes suggested that the experimental procedure to enrich acetylated peptides by using our antibodies was valid. However, we cannot exclude the possibility that our enrichment procedure may not have covered all acetylated peptides present in the cells.

### Succinylome changes in response to the carbon source

In the succinylome analysis, 327 unique succinylated sites on 204 proteins were identified (Table 2). Functional classification analysis showed that largest functional groups contained succinylated proteins involved in translation (34 proteins, 17% of the total), followed by carbohydrate metabolism (28 proteins, 14%) and amino acid metabolism (22 proteins, 11%), similar to the findings reported for the acetylome in this study (Fig 3A). The most heavily succinylated proteins included Icd (8 sites), SrfAB (7 sites), GlyA (6 sites), SucC (6 sites), and Tuf (6 sites).

Pathway enrichment analysis showed that succinylation preferentially targeted the ribosome in the glucose condition (Table 5). In the citrate condition, proteins involved in the citrate cycle and aminoacyl-tRNA biosynthesis as well as the ribosome were preferentially targeted (Table 5). Enrichment of succinylated proteins in the ribosome was also observed in the *E*. *coli* succinylomes [33,36] (see also S4 Table).

**Table 5:** KEGG pathway enrichment for succinylated proteins in the glucose and citrate conditions.

Term^a^	KEGG pathway	Count^b^	%^c^	*p*-value^d^	Fold enrichment^d^	Bonferroni^d^ ^,^ ^e^
***In the glucose condition***						
bsu03010	Ribosome	18	22.2	2.86E–09	5.3	1.12E–07
***In the citrate condition***						
bsu00020	Citrate cycle (TCA cycle)	12	6.0	3.64E–05	4.1	2.25E–03
bsu00970	Aminoacyl-tRNA biosynthesis	12	6.0	5.56E–05	4.0	3.44E–03
bsu03010	Ribosome	22	10.9	2.79E–06	2.9	1.73E–04

^a^KEGG pathway term.

^b^Number of genes matching a given KEGG pathway term.

^c^Percentage of genes matching a given term divided by the total number of input genes in each condition.

^d^The whole genome of *B*. *subtilis* was used as background. (An additional analysis performed using “the total *B*. *subtilis* proteins identified by MS from cell lysates” as background resulted in the same list of enrichment categories).

^e^A Bonferroni cutoff value of 0.05 was used.

Of 327 succinylation sites, 43 sites were reproducibly detected (Table 2), most of which (42 sites) were reproducibly upregulated in the citrate condition (Table 6). The succinylation sites were found in proteins involved in translation (12 sites in FusA, GltX, RplN, RpsC, RpsD, RpsH, RpsK, SerS, and Tuf) and carbohydrate metabolism (13 sites in AcsA, CitZ, Eno, GapA, Icd, LutB, PdhD, Tal, and Zur), consistent with the results of the pathway enrichment analysis (Table 5).

**Table 6:** Succinylation sites (42) upregulated in the citrate condition^a^.

Protein	Description	Position	R-value (glc to cit)^b^	
			Exp. 1	Exp. 2
AcsA	Acetyl-coenzyme A synthetase	K41	C^c^	C
CitZ	Citrate synthase 2	K251	C	C
Eno	Enolase	K232	0.031	C
FabHA	3-Oxoacyl-[acyl-carrier-protein] synthase 3 protein 1	K207	0.042	C
FusA	Elongation factor G	K139	0.030	C
FusA	Elongation factor G	K197	0.048	C
GapA	Glyceraldehyde-3-phosphate dehydrogenase 1	K193	0.075	C
GlnA	Glutamine synthetase	K49	0.042	C
GltX	Glutamate-tRNA ligase	K145	0.034	C
GlyA	Serine hydroxymethyltransferase	K58	0.012	C
GreA	Transcription elongation factor	K55	C	C
GroL	60 kDa chaperonin	K275	0.060	C
HmoA	Heme-degrading monooxygenase	K82	C	C
HupA	DNA-binding protein HU 1	K75	0.074	C
Icd	Isocitrate dehydrogenase	K158	0.024	C
Icd	Isocitrate dehydrogenase	K242	C	C
Icd	Isocitrate dehydrogenase	K59	0.036	C
Icd	Isocitrate dehydrogenase	K70	C	C
LutB	Lactate utilization protein B	K88	C	C
MtnU	Hydrolase	K180	0.021	C
PdhD	Dihydrolipoyl dehydrogenase	K111	0.024	C
PdhD	Dihydrolipoyl dehydrogenase	K380	C	C
PurH	Bifunctional purine biosynthesis protein	K65	0.012	C
RplN	50S ribosomal protein L14	K59	0.046	C
RpoC	DNA-directed RNA polymerase subunit beta'	K638	C	C
RpsC	30S ribosomal protein S3	K106	0.034	C
RpsD	30S ribosomal protein S4	K100	0.066	C
RpsH	30S ribosomal protein S8	K24	C	C
RpsK	30S ribosomal protein S11	K82	0.062	C
SerC	Phosphoserine aminotransferase	K184	C	C
SerS	Serine-tRNA ligase	K84	0.031	C
SrfAA	Surfactin synthase subunit 1	K2579	C	C
TagE	Poly(glycerol-phosphate) alpha-glucosyltransferase	K660	C	C
Tal	Transaldolase	K72	C	C
Tuf	Elongation factor Tu	K178	0.045	C
Tuf	Elongation factor Tu	K308	0.046	0.040
Tuf	Elongation factor Tu	K316	0.024	C
YcgN	1-Pyrroline-5-carboxylate dehydrogenase 2	K23	C	C
YcgN	1-Pyrroline-5-carboxylate dehydrogenase 2	K331	C	C
YdaF	Ribosomal N-acetyltransferase	K36	C	C
YwfI	Heme peroxidase	K154	0.061	C
Zur	Zinc-specific metalloregulatory protein	K20	0.065	C

^a^Succinylation sites that were reproducibly changed in the two experiments were chosen.

^b^R-value (the ratio of glucose to citrate) is shown.

^c^C, detected only in the citrate condition.

Of 327 succinylation sites, 115 sites (35%) overlapped with the acetylation sites found in this study (Fig 3B). This percentage was lower than that in *E*. *coli*, in which 66% of the succinylome overlapped with the acetylome [36]. The overlapping sites were frequently found in proteins associated with the ribosome (17 sites in RplE, RplL, RplM, RplN, RpmA, RpmD, RpsB, RpsC, RpsD, RpsG, RpsH, and RpsP), the citrate cycle (14 sites in CitB, CitZ, Icd, OdhB, SucC, SdhA, and Mdh), and aminoacyl-tRNA biosynthesis (7 sites in ArgS, AsnS, AspS, GatC, GltX, and SerS) (S3 Table). In this study, Icd contained the most overlapping sites (six). Succinylation and acetylation occurred simultaneously at some sites (e.g. K59, K158, K226, K242, K247, and K393 of Icd in the citrate condition), while acetylation and succinylation at other sites switched depending upon the conditions (e.g. K262 of PdhD, acetylated in glucose and succinylated in citrate) (S3 Table). These results suggested that the two acyl modifications mostly targeted different lysine sites and changed at each site in response to the carbon source.

We examined the flanking sequences of the 327 succiyl lysine sites (S4 Fig). Negatively charged residues (D and E) were overrepresented in the regions surrounding these 327 succinyl lysine sites, as observed for the *E*. *coli* succinylome [33]. This result was similar to that observed for the acetyl lysine sites. Comparison of the potential substrate motifs for acetylation and succinylation indicated that the two acyl modifications showed different preferences, i.e., phenylalanine at -2 position in acetyl lysine sites, glutamate at -1 position in acetyl lysine sites, histidine at +1 position in acetyl lysine sites; leucine at +2 position in succinyl lysine sites (S4 Fig).

### Acyl modifications in central carbon metabolism

**Fig 4:**
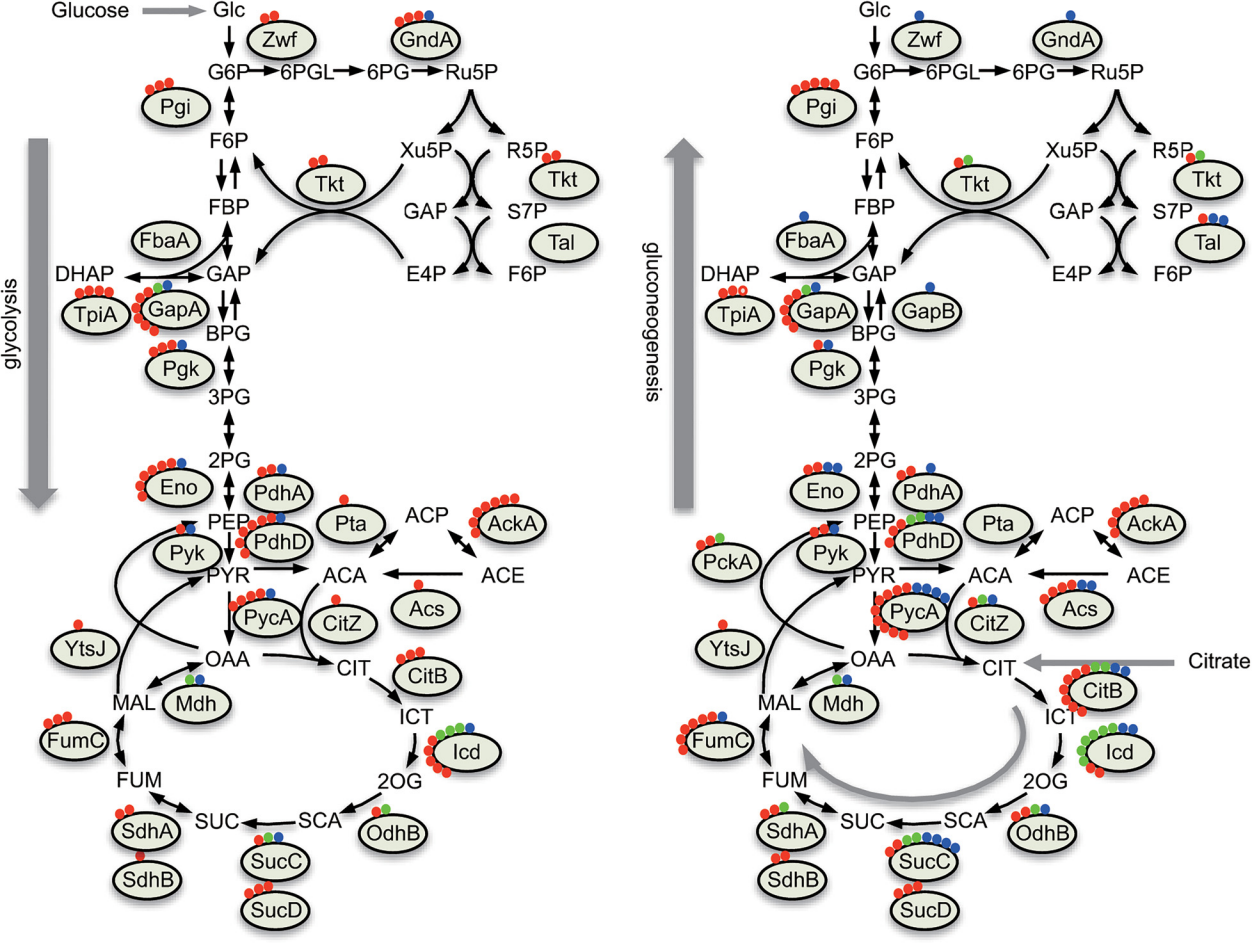
Changes in the acyl modification of central carbon metabolism proteins under glucose or citrate conditions. Red, blue, and green circles represent acetylated, succinylated, and overlapping sites, respectively. The number of circles represents the number of modification sites in each condition. Left, glucose condition; right, citrate condition.

Lysine acetylation frequently occurs in enzymes involved in central carbon metabolism in bacteria and eukarya [5,7,8,11,12,56,57]. Our acetylome analysis confirmed these findings (Fig 4). Acetylation of enzymes involved in glycolysis (GapA, TpiA, Pgk, and Eno) and the pentose phosphate pathway (Zwf and GndA) was more common in the glucose condition than in the citrate condition. In contrast, in the citrate condition, succinylation occurred at more sites and/or was upregulated, especially in proteins associated with the citrate cycle (CitZ, CitB, Icd, OdhB, SucC, SdhA, and FumC) and pyruvate metabolism (PdhA, PdhD, PycA, PckA, and Acs). This observation indicated that acetylation and succinylation of central carbon metabolism proteins changed substantially in response to the carbon source. The correlations between enzyme expression and flux in glycolysis, the pentose phosphate pathway, and pyruvate metabolism are weak [41]. It would be interesting to determine if the acyl modifications found in these pathways have a role in the control of enzymatic activity and/or flux.

### Acyl modifications in RNA polymerase and the ribosome

**Fig 5:**
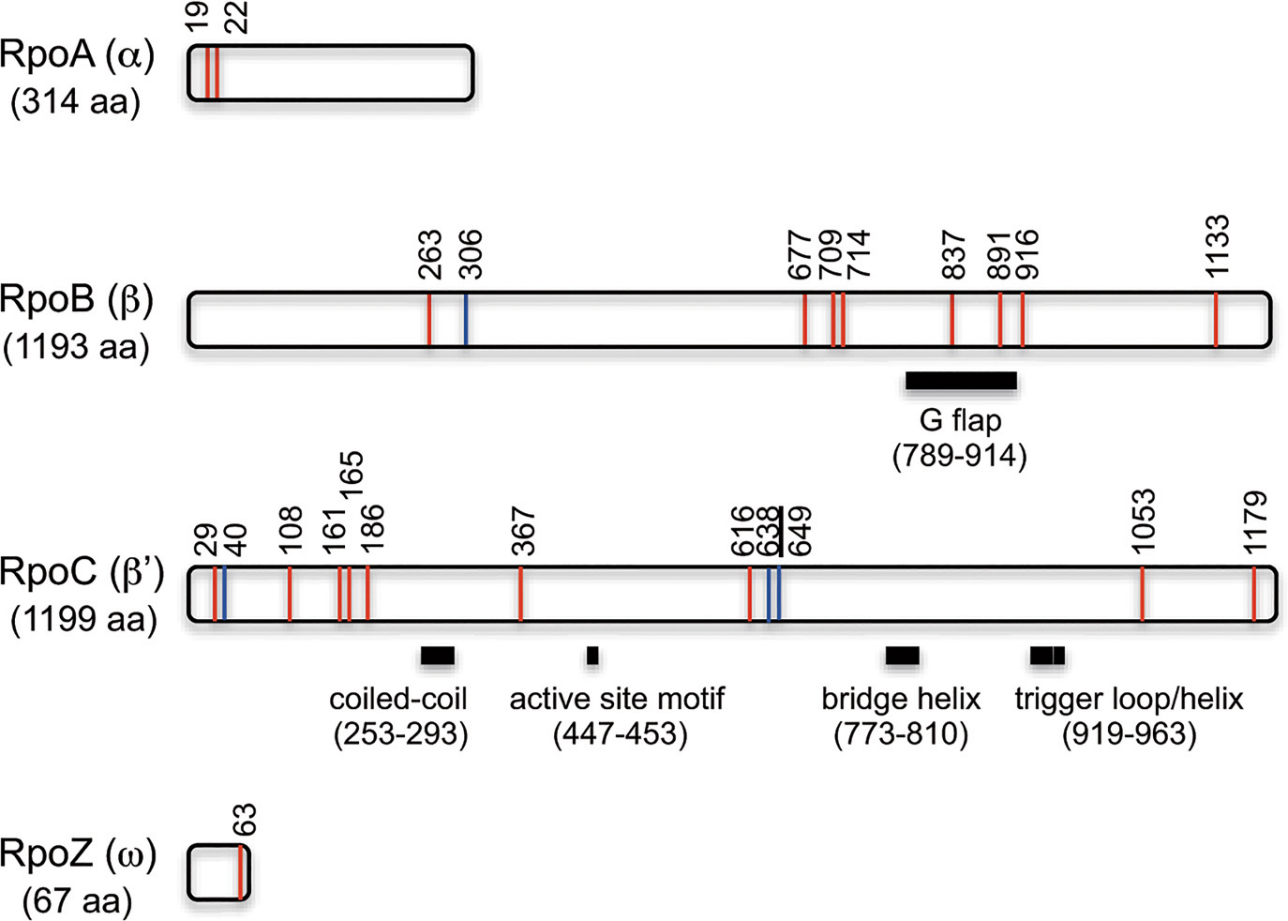
Positions of acyl modification sites in RNA polymerase subunits. Location of acetylation (red line) and succinylation (blue line) sites with amino acid residue numbers are shown. K638, at which succinylation was reproducibly upregulated in the citrate condition, is underlined. Functional and structural regions are shown with black bars; the positions were estimated using amino acid sequence alignment and structural modeling based on information from *E*. *coli* RNAP [60,61].

Acetylation of RNA polymerase (RNAP) has been reported in *E*. *coli* [12,58,59]. In this study, we detected acetylation and succinylation in various *B*. *subtilis* RNAP subunits, including RpoA (α), RpoB (β), RpoC (βʹ), and RpoZ (ω), and sigma factors, including SigA, SigF, and SigH (Fig 5, S2 and S3 Tables). RpoB (β) contained nine acylation sites (eight acetylation sites and one succinylation site). K837, K891, and K916 were located in and close to the G flap region, which undergoes a structural change during holoenzyme formation [60]. RpoC (βʹ) contained 12 acylation sites (nine acetylation sites and three succinylation sites), which were scattered around the structural regions critical for RNAP function (Fig 5). Succinylation at K638 of RpoC was reproducibly upregulated in the citrate condition (Table 6). RpoA (α) and RpoZ (ω) contained two and one acetylation sites, respectively.

Pathway enrichment analysis showed that the ribosome was preferentially targeted by acetylation and succinylation in both glucose and citrate conditions (Tables 3 and 5). Forty-three of the 56 ribosomal proteins were acetylated and/or succinylated (S2 and S3 Tables). Of 140 acylation sites, 103 sites were acetylated, 20 were succinylated, and 17 were shared (see above). The following changes were reproducible: upregulation of acetylation at RplP (K77) and RpsD (K81) in glucose; upregulation of acetylation at RplB (K244 and K252) in citrate; and upregulation of succinylation at RplN (K59), RpsC (K106), RpsD (K100), RpsH (K24), and RpsK (K82) in citrate (Tables 4 and 6). These results indicated that the status of the acyl modifications in RNAP and the ribosome also changed in response to the carbon source.

### The effect of KAT, KDACs, and acetyl-phosphate metabolism on the acyl modifications and growth in *B*. *subtilis*

**Fig 6:**
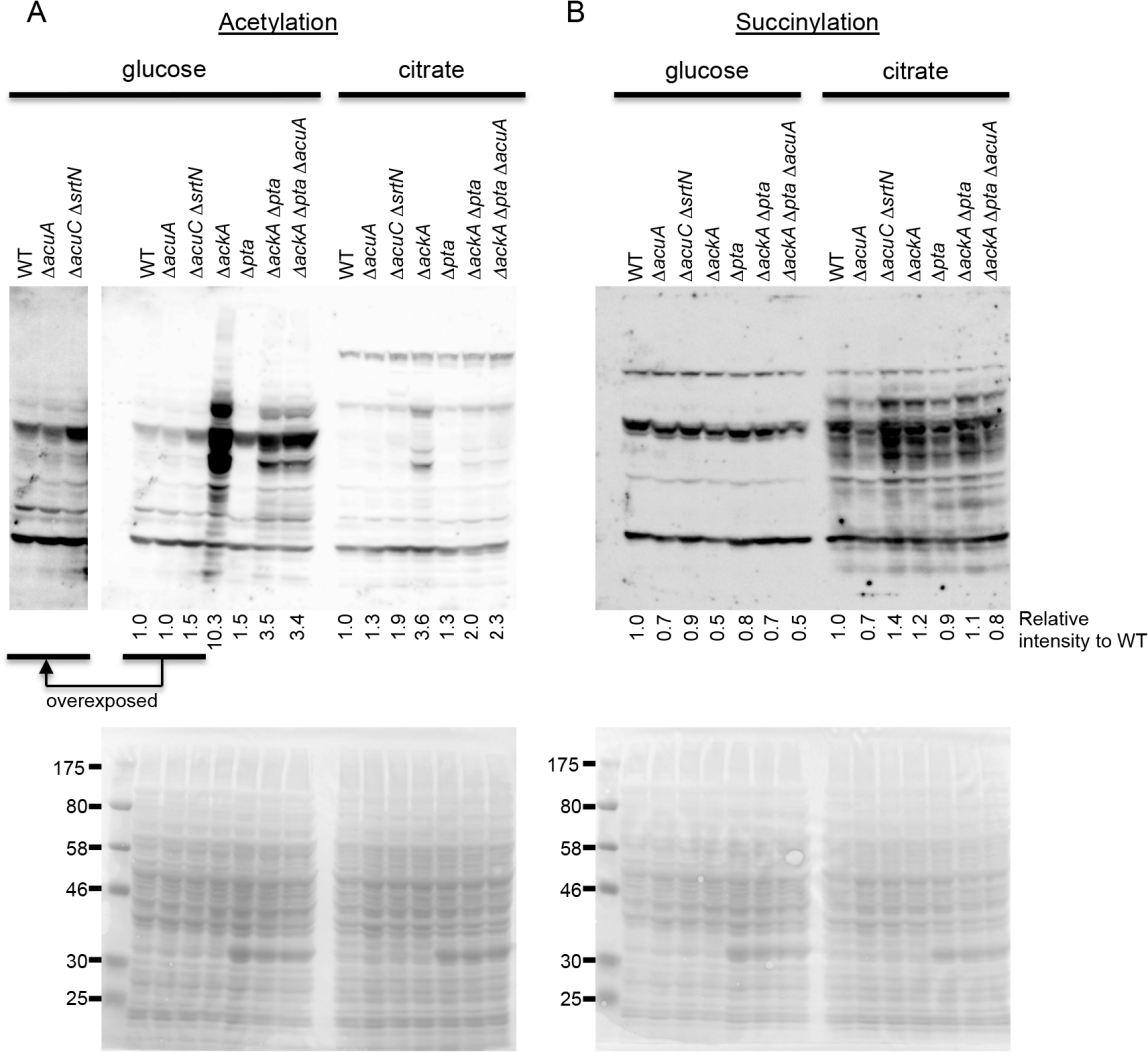
Anti-acetyl lysine and anti-succinyl lysine western blot analyses. 168 (WT), SS110 (Δ*acuA*), SS38 (Δ*acuC* Δ*srtN*), SS51 (Δ*ackA*), SS52 (Δ*pta*), SS53 (Δ*ackA* Δ*pta*), and SS111 (Δ*ackA* Δ*pta* Δ*acuA*) strains were grown in modified Spizizen’s minimal medium supplemented 30 mM glucose or citrate. Cells were harvested when the OD_660_ reached 0.5. Total lysates containing 20 μg of protein were separated by 10% SDS-PAGE. The signal intensity in each lane was quantified and was expressed relative to the value for the wild-type strain after subtracting the background. Upper panel: western blot with anti-acetyl lysine (A) and anti-succinyl lysine (B) antibodies; Lower panel: Ponceau staining.

*B*. *subtilis* possesses one KAT, AcuA, and two KDACs, AcuC and SrtN. *B*. *subtilis* also has phosphotransacetylase (Pta) and acetate kinase (AckA) involved in the generation of acetyl-P, which is utilized for non-enzymatic acetylation. To examine the extent to which these factors contribute to lysine acetylation and succinylation in *B*. *subtilis*, we performed western blot analysis using the wild type strain and isogenic mutants that lack KAT (Δ*acuA*), lack both KDACs (Δ*acuC* Δ*srtN*), accumulate acetyl-P (Δ*ackA*), or block acetyl-P production (Δ*pta*, Δ*ackA* Δ*pta*, or Δ*ackA* Δ*pta* Δ*acuA*). We quantified the signal intensity in each lane and expressed it relative to the wild-type strain (Fig 6). In the minimal glucose condition, strain SS110 (Δ*acuA*) showed a negligible change in the acetylation level when compared to the wild type strain, indicating that the KAT has a small effect on global acetylation, as also observed in *E*. *coli* [12,26]. The SS38 (Δ*acuC* Δ*srtN*) strain showed a 1.5-fold increase in total acetylation compared to the wild-type strain, which was reproducible in four repeated experiments (data not shown). Global acetylation increased dramatically (10.3-fold) in strain SS51 (Δ*ackA*), as previously observed in *E*. *coli* [9,12,26]. Acetylation in strains SS52 (Δ*pta*, 1.5-fold), SS53 (Δ*ackA* Δ*pta*, 3.5-fold), and SS111 (Δ*ackA* Δ*pta* Δ*acuA*, 3.4-fold) was higher than in the wild type strain, but lower than in strain SS51 (Δ*ackA*). In these strains, a block in acetyl-P production might cause acetyl-CoA to accumulate and enhance acetylation. Acetylation in the SS111 (Δ*ackA* Δ*pta* Δ*acuA*) strain was comparable to that in the SS53 (Δ*ackA* Δ*pta*) strain, suggesting that it might be induced by KATs other than AcuA or through the non-enzymatic mechanism by using acetyl-CoA as observed in the mitochondria [25] Increased acetylation in the SS53 strain compared with that in the wild-type strain was different from that observed in the Δ*ackA* Δ*pta* double mutant of *E*. *coli*, which exhibited lower acetylation than the wild-type strain [12]. In the wild type and mutant strains, acetylation levels were lower in the minimal citrate condition than in the minimal glucose condition (Fig 6A).

In contrast, in the wild type and mutant strains, succinylation levels were higher in the citrate condition than in the glucose condition, consistent with the results of Fig 1. Succinylation level in all the mutant strains, except SS38 (Δ*acuC* Δ*srtN*), was comparable to that in the wild-type strain (Fig 6B). The SS38 (Δ*acuC* Δ*srtN*) strain showed a reproducible increase (1.4-fold) in succinylation compared to the wild-type strain in the presence of citrate. Other changes apparently observed in Fig 6B could not be reproduced in four repeated western blot analyses.

We also examined the growth curves of the mutant strains. The growth of strains SS52 (Δ*pta*), SS53 (Δ*ackA* Δ*pta*), and SS111 (Δ*ackA* Δ*pta* Δ*acuA*), in which production of acetyl-P was blocked, was slightly but reproducibly better than that of the wild type strain on glucose in the late exponential phase (Fig 2C). These mutants did not show a growth advantage on citrate (Fig 2B). The growth of strain SS110 (Δ*acuA*) was slightly but reproducibly better than that of the wild type strain on both glucose and citrate (Fig 2C and 2D). These results suggest that modulation of protein acylation affected *B*. *subtilis* growth. Glucose-dependent growth inhibition of the *ackA* mutant in a rich medium (TSS medium containing 1% casamino acids) has been reported previously [62]. Moreover, the *ackA* and *pta* mutants do not show substantial growth defects in the presence of glucose in CSK medium (C minimal medium containing potassium succinate and potassium glutamate) [63]. We also observed growth defects in the *ackA* mutant (SS51) compared to the wild-type strain grown in L medium (data not shown), which was similar to those reported in *E*. *coli* [64]. However, no effect was observed when the strains were grown in a minimal glucose medium (Fig 2), which was consistent with the results of previous studies. These results suggest that rapid and/or excessive accumulation of acetyl-P in the rich medium exerted harmful effects on the cells, but gradual accumulation of acetyl-P did not.

## Discussion

In this study, we investigated changes in lysine acetylation and succinylation in *B*. *subtilis* in response to the carbon source. We performed acetylome and succinylome analyses using the same cells and experimental conditions, enabling us to analyze the precise relationship between the two modifications. Global lysine acetylation and succinylation exhibited a reciprocal pattern during growth in glucose and citrate conditions; acetylation was high in the glucose condition, whereas succinylation was high in the citrate condition (Figs 1 and 6). This pattern of acetylation and succinylation is likely linked to the metabolic states in the glucose and citrate conditions, in which acetyl-CoA and acetyl-P are produced by glycolysis and succinyl-CoA is produced by the citrate cycle. A similar observation has been reported in *E*. *coli*, in which exposure to glucose increased lysine acetylation [9,12]. In *E*. *coli*, glucose supplementation in M9 medium increased lysine succinylation [33]. However, we did not observe high succinylation in *B*. *subtilis* in glucose-supplemented minimal medium condition (Fig 1). Therefore, the mechanisms that induce lysine succinylation in *E*. *coli* and *B*. *subtilis* might be different, at least under the glucose condition.

We evaluated changes in acyl modifications by assessing the R-value, which distinguishes changes in the percentage of acylated peptides in the peptide population from changes in acylated peptides proportional to the protein abundance. We detected acyl modification sites whose percentage reproducibly increased in either growth condition (Tables 4 and 6). Meanwhile, one-third of the 482 quantifiable acetylation sites changed in proportion to the protein abundance (Table 2). Acetylation and succinylation targeted proteins in similar categories, but a substantial portion (65%) of the succinylome did not overlap with the acetylome (Fig 3B). Furthermore, the substrate motifs for lysine acetylation and succinylation were slightly, but distinctly, different (S4 Fig). Based on these observations, we postulated that lysine acetylation and succinylation may depend on different factors such as (i) chemical modifications depending on different molecular characteristics of the acyl donor (size and/or charge), (ii) different molecular environments around the lysine substrate, or (iii) different modifying enzymes.

Succinylation at almost all sites was increased in the citrate condition, likely reflecting the increased concentration of succinyl-CoA. No enzyme that catalyzes lysine succinylation has been identified in any organisms, including *B*. *subtilis*. At present, we do not know whether AcuA, the only known KAT in *B*. *subtilis*, also catalyzes lysine succinylation, or if a yet-to-be-identified KAT functions as a succinyltransferase. *B*. *subtilis* possesses more than 50 proteins with GNAT motifs, some of which might be KATs. Recent *in vitro* studies have shown that non-enzymatic lysine succinylation occurs at lower (micromolar) concentrations of succinyl-CoA than non-enzymatic acetylation, which occurs at millimolar concentrations [25,36]. Therefore, we speculate that a portion of the increased succinylation observed in the citrate condition occurred non-enzymatically.

We observed changes in the acyl modification of proteins in the central carbon metabolic pathways when cells were cultured in minimal glucose or citrate conditions, in which carbon fluxes are different (Fig 5). Wang *et al*. [5] have suggested that reversible lysine acetylation functions as a regulatory mechanism to coordinate metabolic fluxes. Chubukov *et al*. [41] have recently shown in *B*. *subtilis* that the correlation between flux and enzyme concentration is low in central metabolic pathways, especially in the glycolysis pathway, pentose phosphate pathway, and anaplerotic pathway. Thus, the changes in lysine acetylation and succinylation found in this study might explain metabolic flux changes in response to carbon sources, as suggested by Chubukov *et al*. Further study is needed to examine the roles of acyl modifications in metabolic enzyme regulation and determine how acyl modifications coordinate metabolic fluxes in response to carbon sources.

We showed that mutations that likely modulate protein acylation affected *B*. *subtilis* growth (Fig 2). Mutations that block acetyl-P production (and probably result in acetyl-CoA accumulation) positively affected growth on glucose. The Δ*acuA* (KAT-deficient) mutation slightly but reproducibly enhanced growth on citrate. These results are similar to those reported in *Salmonella enterica*, in which increased and decreased acetylation caused by disruption of CobB (KDAC) and Pat (KAT) enhanced growth on glucose and citrate, respectively [5]. At present we do not have a reasonable explanation for that the Δ*acuA* mutant showed a better growth in both minimal glucose and citrate conditions. Although only a negligible change in the acylation status was detected in the Δ*acuA* mutant with western blot analysis (Fig 6), future studies will use MS-based analysis to identify minor but functional acetylation and/or succinylation sites.

Increased acetylation in the SS53 (Δ*ackA* Δ*pta*) strain compared to that in the wild-type strain observed in the present study was different from that observed in the Δ*ackA* Δ*pta* double mutant of *E*. *coli*, which exhibited lower acetylation than the wild-type strain [12]. We currently consider that acetyl-CoA and not acetyl-P mainly contributes to the acetylation in this Δ*ackA* Δ*pta* double mutant either in a KAT-dependent or in a non-enzymatic manner. Another possibility is the presence of currently unidentified Pta-independent pathway(s) to produce acetyl-P in *B*. *subtilis*. According to the KEGG database, YdaP might be such a candidate. YdaP is a homologue of *E*. *coli* pyruvate dehydrogenase (quinone) PoxB, which directly converts pyruvate to acetate [65,66]. However, recent genetic data suggest that *Streptococcus pneumoniae* SpxB, a homologue of *B*. *subtilis* YdaP, is involved in the production of acetyl-P [67,68]. Further studies are necessary to determine the donor that increases acetylation in the Δ*ackA* Δ*pta* mutant of *B*. *subtilis*. We observed a slight but reproducible increase in acetylation and succinylation in the SS38 (Δ*acuC* Δ*srtN*) strain (Fig 6A and 6B). It may be considered that either or both of the two KDACs catalyze deacetylation and desuccinylation as reported with the *E*. *coli* CobB [33]. We need further studies to determine the possibility.

We also found changes in acyl modifications in RNAP and the ribosome; these changes might be involved in novel transcriptional and translational regulation mechanisms in response to nutrient signals. Lysine acetylation in RNAP is involved in glucose and growth factor-induced transcription in *E*. *coli* and mouse, respectively [58,59,69]. It will be interesting to determine how protein activity and function are regulated by different acyl modifications and whether there is cross talk between acetylation and succinylation. Together with recent advances in our understanding of enzyme regulation by acyl modifications [5,20,24,40,55,58,59,70], our results suggest that acetylation and succinylation are involved in the physiological responses and adaptations to changes in carbon nutrients. Although more studies are needed to determine the exact roles of the acyl modifications detected in this study, the results presented here provide a foundation for exploring the important and influential roles of acyl modifications in *B*. *subtilis*.

## Supporting Information

S1 Fig Dot blot analysis to determine the specificity of anti-acetyl lysine and anti-succinyl lysine antibodies. Serial 10-fold dilutions of BSA (2.0 μg), acetylated BSA (2.2 μg), and succinylated BSA (1.4 μg) were blotted onto a PVDF membrane. The blot was incubated with anti-acetyl lysine or anti-succinyl lysine antibody (1:1000 dilution in 3% milk-TBST).
(TIF)

S2 Fig Western blot analysis of the wild type 168 strain and its lysine auxotroph derivative TM61. Cells were grown in modified Spizizen’s minimal medium supplemented with 30 mM glucose or citrate as the sole carbon source. Total lysates containing 20 μg of protein were separated by 10% SDS-PAGE. Left: Ponceau staining; right: western blot with anti-acetyllysine (A) and anti-succinyllysine (B) antibodies.
(TIF)

S3 Fig Venn diagram showing the number of acetylation and succinylation sites identified in this study. The number of total unique acetylation (upper) and succinylation (lower) sites identified in exp. 1 (glucose-heavy labeling, black circle) and in exp. 2 (citrate-heavy labeling, grey circle) is indicated in parentheses.
(TIF)

S4 Fig Analysis of lysine acetylation and succinylation motifs by using the IceLogo (A) and Motif-X (B) algorithms. **(**A) A consensus sequence logo of -10 to +10 positions relative to the acetylation (left) and succinylation (right) sites was generated using iceLogo. The frequencies are shown as percentage differences (*p* = 0.05). (B) Sequence motifs surrounding the modification sites were analyzed using motif-X. The parameters were as follows: width, 13 residues (6 amino acids on each side of a modification site); occurrence threshold, 20; *p*-value threshold, 0.000001 for acetylation and 0.0001 for succinylation; and background, unaligned motif data.
(TIF)

S1 Table Oligonucleotide primers used in this study. (PDF)

S2 Table A comprehensive list of unique acetylation and succinylation sites identified in this study. The sheet “exp1_Ac (956)” shows a list of 956 unique acetylation sites identified in exp. 1. “Exp1_Sc (271)” shows a list of 271 unique succinylation sites identified in exp. 1. “Exp2_Ac (886)” shows a list of 886 unique acetylation sites identified in exp. 2. “Exp2_Sc (100)” shows a list of 100 succinylation sites identified in exp. 2. Information regarding protein accession number, protein description, protein functional classification based on the KEGG pathway database, gene name, position of the modification and its surrounding sequence, peptide peak ratio (heavy/light [pep]), protein ratio (heavy/light [pro]), and R-value is provided. “H” and “L” indicate that the peptide was detected only in the heavy or light sample in each experiment, respectively.
(XLSX)

S3 Table A merged list of all acetylated, succinylated, and overlapping sites identified in this study. Peptide peak ratios (glucose to citrate) and R-values are shown. “G” and “C” indicate that the peptide was detected only in glucose or citrate in each experiment, respectively. The data was derived from S2 Table.
(XLSX)

S4 Table KEGG pathway enrichment for acetylated and succinylated proteins in *E*. *coli* in minimal glucose conditions. The acetylome and succinylome datasets obtained from Weinert *et al*. [9,36] were analyzed with DAVID using the same conditions used in Tables 3 and 5.
(XLSX)
