# Supplementary figures and images for: Changes in the Acetylome and Succinylome of Bacillus subtilis in Response to Carbon Source
Source: PLoS One. 2015 Jun 22;10(6):e0131169. doi: 10.1371/journal.pone.0131169 (PMC4476798; doi:10.1371/journal.pone.0131169)

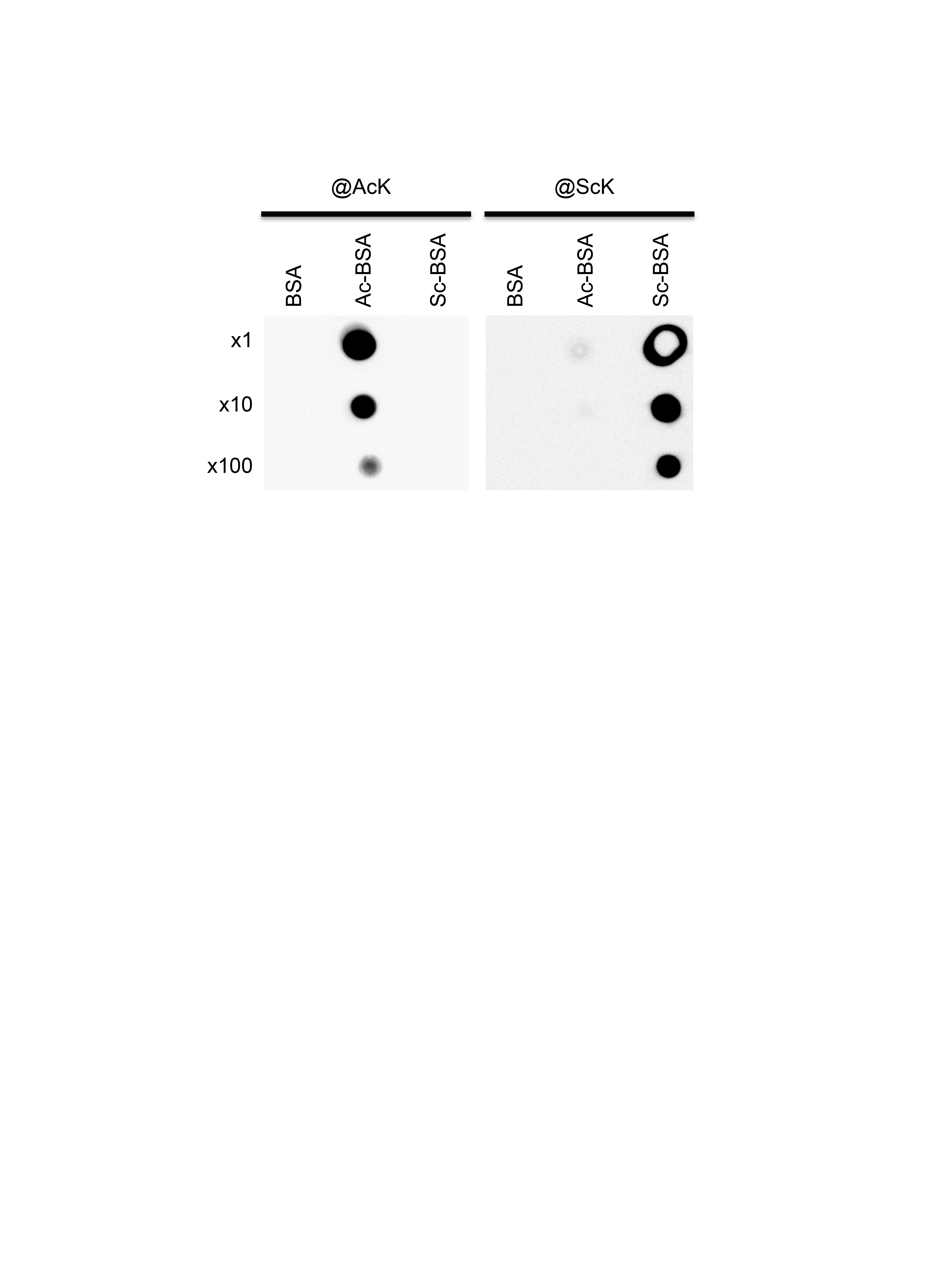

Supplement: S1 Fig — Serial 10-fold dilutions of BSA (2.0 μg), acetylated BSA (2.2 μg), and succinylated BSA (1.4 μg) were blotted onto a PVDF membrane. The blot was incubated with anti-acetyl lysine or anti-succinyl lysine antibody (1:1000 dilution in 3% milk-TBST). (TIF) [file pone.0131169.s001.tif]

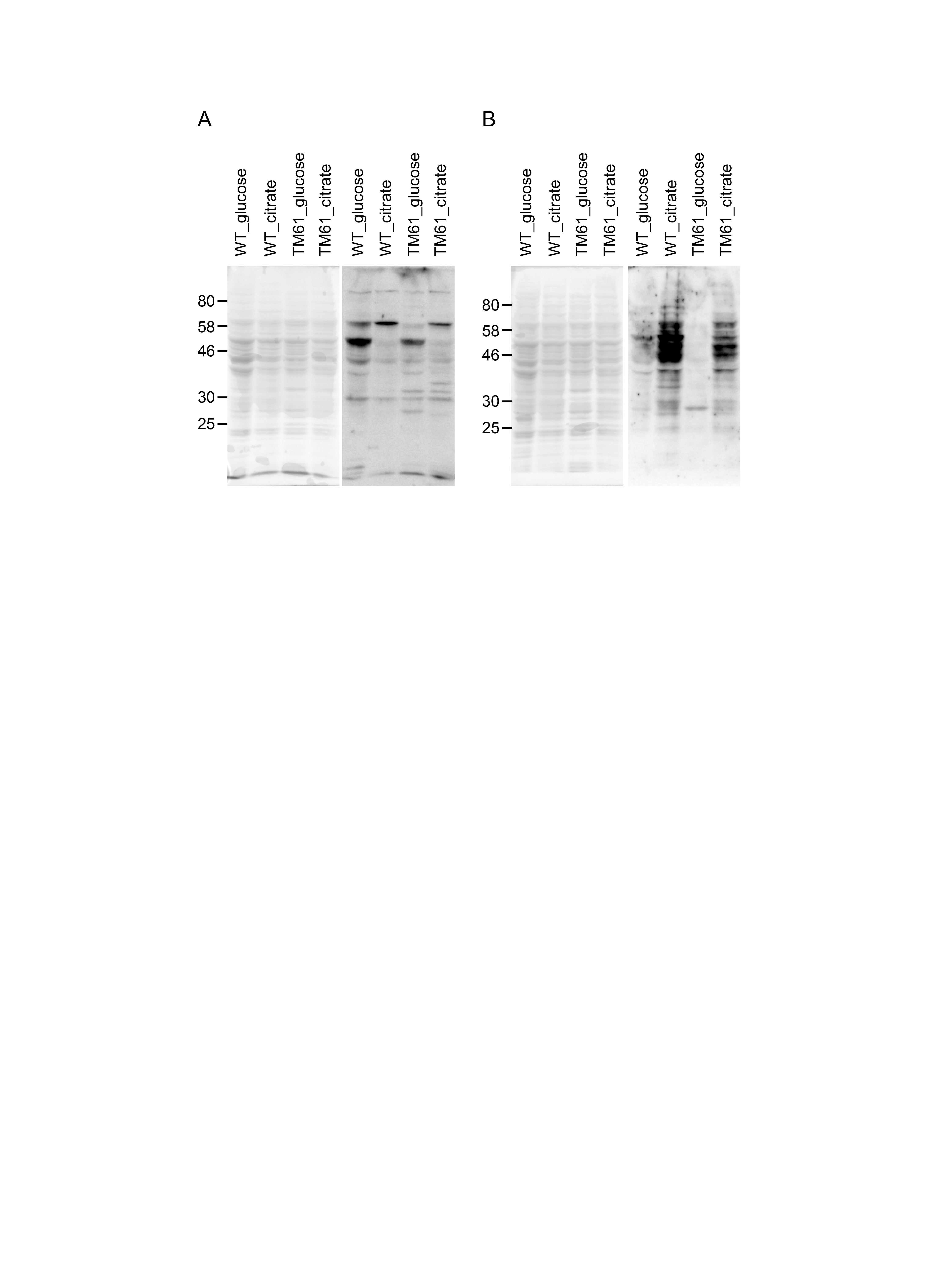

Supplement: S2 Fig — Cells were grown in modified Spizizen’s minimal medium supplemented with 30 mM glucose or citrate as the sole carbon source. Total lysates containing 20 μg of protein were separated by 10% SDS-PAGE. Left: Ponceau staining; right: western blot with anti-acetyllysine (A) and anti-succinyllysine (B) antibodies. (TIF) [file pone.0131169.s002.tif]

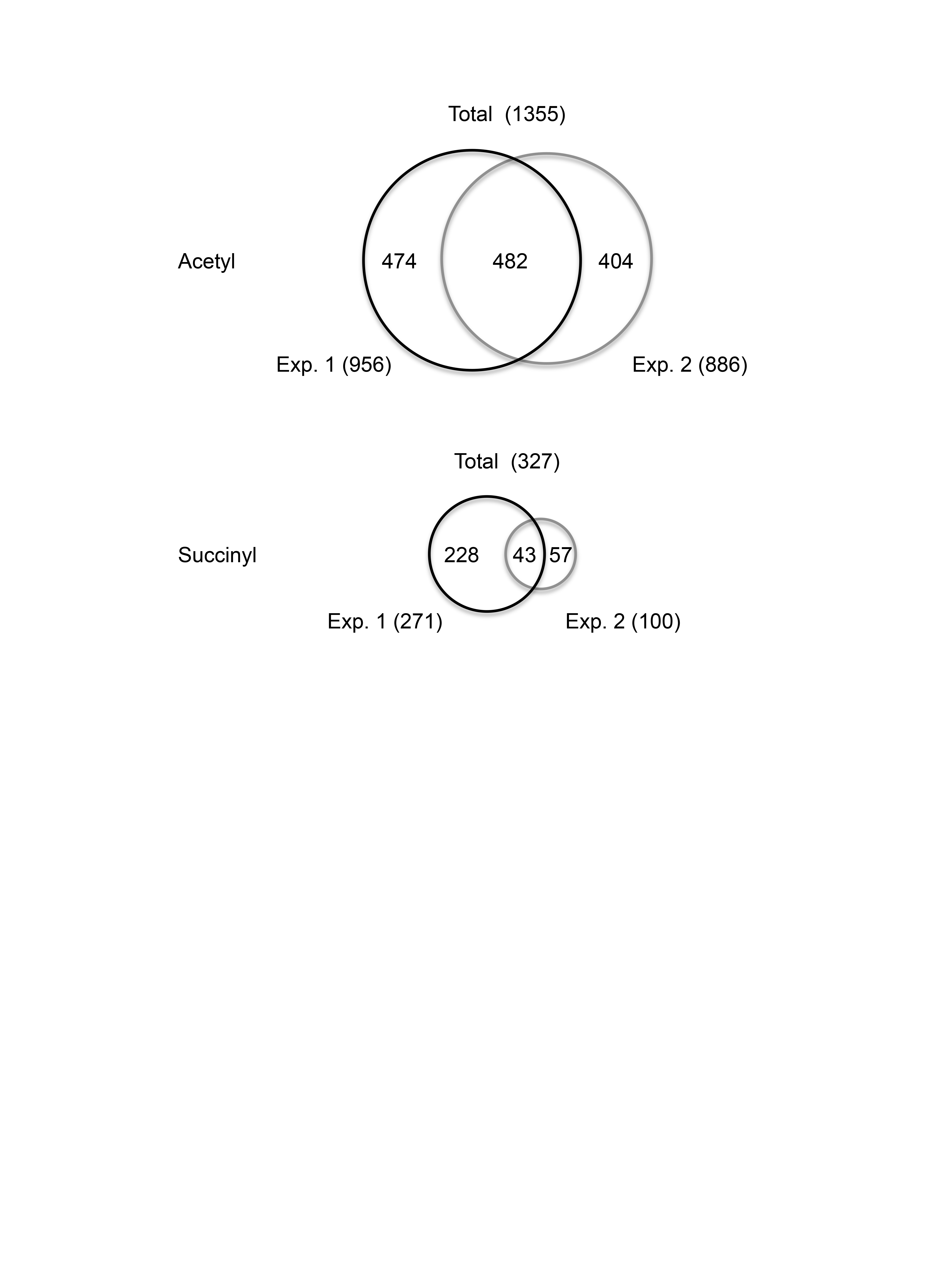

Supplement: S3 Fig — The number of total unique acetylation (upper) and succinylation (lower) sites identified in exp. 1 (glucose-heavy labeling, black circle) and in exp. 2 (citrate-heavy labeling, grey circle) is indicated in parentheses. (TIF) [file pone.0131169.s003.tif]

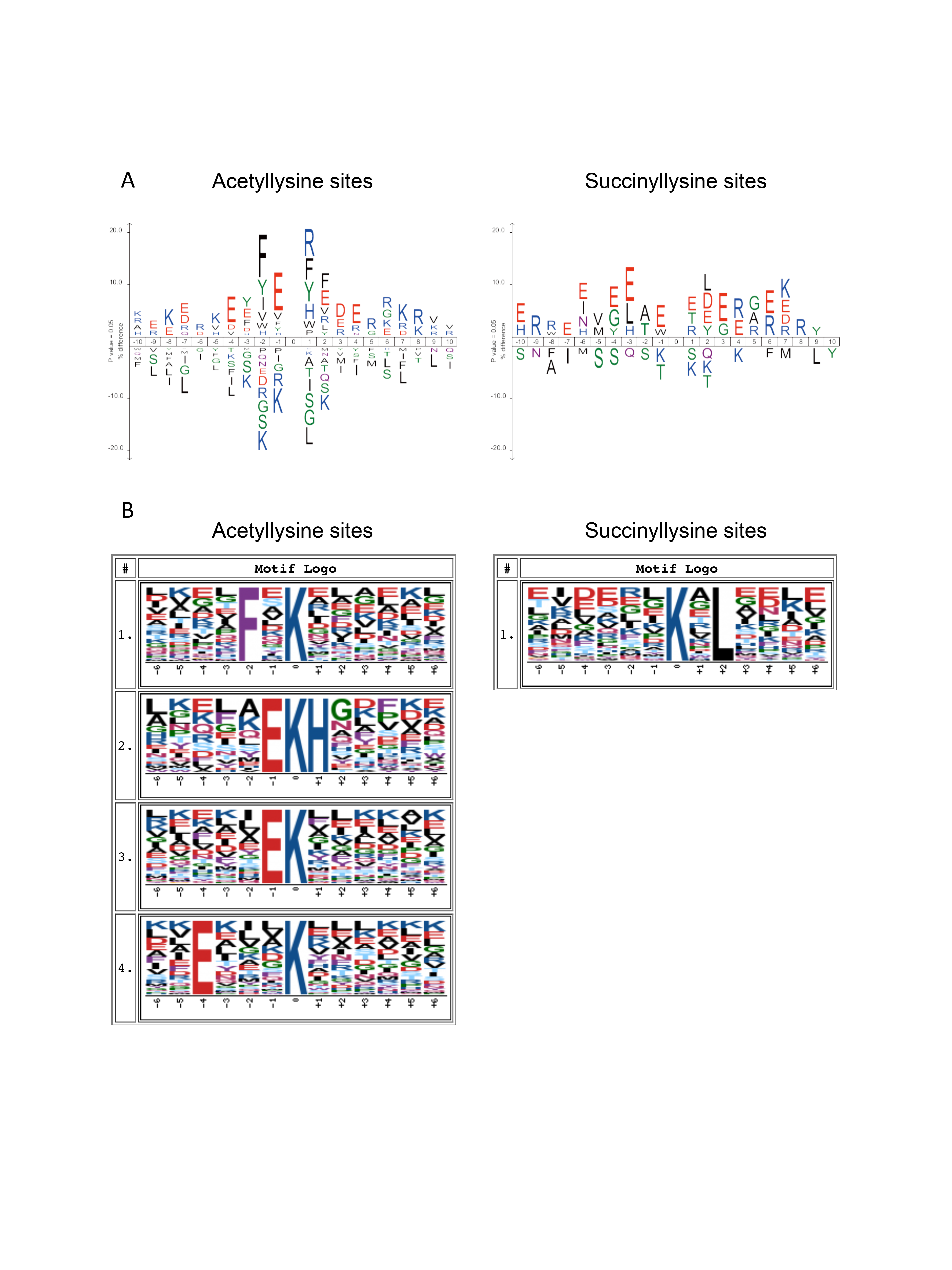

Supplement: S4 Fig — (A) A consensus sequence logo of -10 to +10 positions relative to the acetylation (left) and succinylation (right) sites was generated using iceLogo. The frequencies are shown as percentage differences (p = 0.05). (B) Sequence motifs surrounding the modification sites were analyzed using motif-X. The parameters were as follows: width, 13 residues (6 amino acids on each side of a modification site); occurrence threshold, 20; p-value threshold, 0.000001 for acetylation and 0.0001 for succinylation; and background, unaligned motif data. (TIF) [file pone.0131169.s004.tif]
