# Supplementary material for: Changes in the Acetylome and Succinylome of Bacillus subtilis in Response to Carbon Source
Source: PLoS One. 2015 Jun 22;10(6):e0131169. doi: 10.1371/journal.pone.0131169 (PMC4476798; doi:10.1371/journal.pone.0131169)
Supplement: S1 Table — (PDF) [file pone.0131169.s005.pdf]

S1 Table. Oligonucleotide primers used in this study.

Primer	Sequence
lysAmut1-F	TAACCTATGTCGCAACCCATCCGT
lysAmut1-R	CCATGTTGATTTTATCAGCTTGTGCCGT
lysAmut2-F	ACGGCACAAGCTGATAAAATCAACATGG
lysAmut2-R	AAGTTTACGCTAATCAACTCTAGCAC
trpC2hisC-F	AGCGGAGCATTACATGCTCGTTGA
trpC2hisC-R	TTCGCAATGCTCTCATCTCCGGCT
acuAdel1-F	GAATTCGCCTCACTGTTTTCAAGCCGG
acuAdel1-R	GGATCCTTCCACTAATTCACCGTCCCA
acuAdel2-F	CTGCAGTATTAACTGACACTGACAAAG
acuAdel2-R	GCATGCCATTTCTTTGTAATGCCTGCA
acuCdel1-F	GGTACCAGACGCTCCTGACCGTCAGC
acuCdel1-R	TCTAGAGTCTCTCATAGCAGATCCCT
acuCdel2-F	TCTAGAAGAACAAAGTAAAAAACGAC
acuCdel2-R	CTGCAGTCATCAAGCAGGTAATGGTG
srtNdel1-F	GGTACCCGCTCACTGATTGTGGCGCT
srtNdel1-R	TCTAGATGTTTCCAACTCAACACCGC
srtNdel2-F	TCTAGAATGAAAAAGTGAGCCGCTTT
srtNdel2-R	AAGCTTCATCTTCATAAGCTGCCTGC
ptadel1-F	CGAAAGGTGATTGCCGACAAAGAATGAACC
ptadel1-R	GGTCATGCTTGTGTCAATAAAACCTCCTCAAAAAG
ptadel2-F	ATTTTGAACGATGACCTAAAATTGAAGACAATGGC
ptadel2-R	GTATTCTCCATGAGAGGAGAATGAATGTAAG
pta_kan-F	GACACAAGCATGACCATTATGACTAG
pta_kan-R	GGTCATCGTTCAAAATGGTATGC
ackAdel1-F	GCGGACAAAAAGGAACTGACCATTC
ackAdel1-R	TACAGATCGATCCTCTAGAGGATTGACGCTCCTTTATACT

ackA_spc-2F	CTCTAGAGGATCGATCTGTATAATA
ackA_spc-2R	CTAATTGAGAGAAGTTTCTATAGAA
ackAdel2-F	TAGAAACTTCTCTCAATTAGATCGCATGAAAGCACATTCT
ackAdel2-R	TGTGGGCCTGCAAAGAGATAAGCGC

a) mutation sites in the *lysA* gene are shown with underlined.
